# The prevalence of masked hypertension in patients with lone atrial fibrillation: a cross sectional analytical study

**DOI:** 10.1038/s41598-023-36853-3

**Published:** 2023-06-16

**Authors:** Irit Ayalon-Dangur, Shachaf Ofer-Shiber, Tzippy Shochat, Irina Genin, Maya Arlyuk, Alon Grossman

**Affiliations:** 1 Department of Internal Medicine E, Rabin Medical Center, Petah Tikva, Israel; 2 Sackler Faculty of Medicine, Tel Aviv University, Tel Aviv, Israel; 3 Department of Rheumatology, Rabin Medical Center, Petach Tikva, Israel; 4 The Department of Emergency Medicine, Rabin Medical Center, Beilinson Campus, Israel; 5 Bio-Statistical Consultant, Rabin Medical Center, Beilinson Campus, Israel; 6 Department of Internal Medicine B, Rabin Medical Center, Petah Tikva, Israel

**Keywords:** Cardiology, Endocrinology, Nephrology, Risk factors

## Abstract

Atrial fibrillation (AF) is prevalent in individuals with essential hypertension (HTN). Masked hypertension occurs in up to 15% of the general population and is associated with adverse clinical outcome. The aim of the current study was to evaluate the prevalence of masked hypertension in apparently normotensive individuals with lone AF. A cross sectional analytical study performed at the Rabin Medical Center included all patients > 18 years who visited the emergency department (ED) in the years 2018–2021 with idiopathic AF, had normal blood pressure (BP) values during their ED visit and did not have a history of hypertension or current use of anti-hypertensives. Ambulatory blood pressure monitoring (ABPM) was performed in all eligible patients within 30 days from ED visit. Data collected included information from the ED visit and data extracted from the monitoring device. A total of 1258 patients were screened for eligibility, of which 40 were included in the analysis. The average age was 53.4 ± 16 years, 28 patients (70%) were males. Overall, 18 individuals (46%) had abnormal BP values according to the 2017 ACC/AHA guidelines for the diagnosis of hypertension. Of these, 12 had abnormal 24-h BP average (≥ 125/75 mmHg), one had isolated daytime abnormal average (≥ 130/80 mmHg) and 11 had isolated night time abnormal average (≥ 110/65 mmHg). Masked hypertension is prevalent in patients with lone AF without a diagnosis of HTN and performing ABPM in such individuals should be strongly considered.

## Introduction

Atrial fibrillation (AF) is prevalent in individuals with essential hypertension^1–4^ and the degree of control of blood pressure is closely associated with the incidence of AF^5^. Masked hypertension is a condition in which blood pressure is within normal limits during office measurements but is elevated on ambulatory blood pressure monitoring. This condition is reported to occur in up to 30% of hypertensives^6,7^ and is associated with similar adverse clinical outcomes as hypertension diagnosed on office measurements^8–10^. Masked hypertension has been reported to affect atrial remodeling^11^ but its association with the occurrence of atrial fibrillation has not been reported. Although the reliability of ambulatory blood pressure monitoring (ABPM) for the diagnosis of hypertension in patients with AF has been questioned^12^, it is still a legitimate method for diagnosing hypertension in patients with AF. The prevalence of masked hypertension in the general population is about 10–15%^6,7,13^. Different studies have found that the prevalence is similar in different populations. In the American population the estimated prevalence of masked hypertension in the years 2005–2010 was 12.3%^6^. In Israel a study published in 2005 reported 11% prevalence of masked hypertension^14^.

The aim of the current study is to evaluate the prevalence of masked hypertension in individuals with lone AF in order to determine whether an active search for masked hypertension should be performed in those with lone AF.

## Methods

### Study design

This study was a cross sectional analytical study, single center, non-interventional study performed in the Rabin Medical Center emergency department (ED), one of the largest ED in Israel.

### Study population

Patients > 18 years old with lone AF who presented to the Beilinson hospital emergency department (ED) from March 2018 to June 2021 were identified. All patients with a previous diagnosis of hypertension were excluded as well as patients chronically treated with anti-hypertensive medications such as Angiotensin-converting-enzyme inhibitors (ACEi), Angiotensin receptor blockers (ARBs), diuretics, nitrates, alpha and beta blockers and other drugs that may affect blood pressure.

Patients in which BP measurements were higher than 140/90 mmHg (or stage 2 hypertension based on the ACC/AHA guideline) during the ED visits were excluded. We chose to include individuals with BP 130/80–140/90 mm Hg given that non-office-based measurement may reasonably be higher in the ED. However, in addition to the main analysis, we also performed an additional analysis in which patients with stage 1 hypertension were also excluded.

Blood pressure measurement in the emergency department was performed using PHILIPS SureSigns VSi blood pressure monitoring device, which is an automatic electronic device.

The diagnosis of AF was based on 12-lead ECG performed in the emergency department (ED). Lone AF was defined as AF without structural heart disease or other condition triggering the event, and therefore patients with AF attributable to cardiac disease, pulmonary disease, metabolic/endocrine conditions, medications, substance abuse, anemia, alcohol, electrolyte abnormalities or acid/base disorders were excluded. Pregnant women were also excluded and patients who were treated for AF in the ED with agents such as calcium channel blockers or beta blockers (excluding sotalol) were excluded due to the possible effect of these medications on BP values. Patients treated with antiarrhythmic therapy during the ED visit, which included mainly amiodarone and propafenone were included.

### Ambulatory BP measurement and data collection

Patients who fulfilled eligibility criteria underwent 24-h ambulatory blood pressure monitoring (ABPM) within 30 days from ED visit, regardless of the decision to hospitalize or discharge the patient. The monitoring was performed using Oscar 2™ Ambulatory Blood Pressure Monitor from SunTech Medical®. Prior to the examination, a resting ECG was performed in all patients. If elevated BP values was identified, the primary care physician was notified to continue follow-up and treatment accordingly. From the screening process until the 24 h ABPM examination the patient continued to take his regular medications as well as medication prescribed due to the new diagnosis of AF (e.g. anticoagulants). The study information was collected from different sources such as the medical records, the hospital visit during the connection to the device and from extraction of the output of the “Holter” examination. The data collected included information from the admission such as demographic variables, co-morbidities, vital signs, ECG results, symptoms and laboratory on admission, medications, hospitalization or discharge information and the medications prescribed for the treatment of AF. The data regarding the 24 h ABPM output was 24 h, daytime and nighttime blood pressure measurements, duration of examination, symptoms during the examination and any special activity of the patient during the test. The definition of masked hypertension in this study was any abnormal blood pressure measurement according to the 2017 ACC/AHA guidelines for the diagnosis of hypertension^15^ during the ABPM examination.

Furthermore, as part of the ABPM examination, a calculation of %dipping was performed, and was defined as the proportional reduction in nighttime BP compared with daytime BP. Non-dippers were patients whose BP failed to decrease by at least 10% during sleep. A calculation of systolic and diastolic BP load during 24 h, daytime and nighttime, defined as the proportion of time that the systolic and diastolic BP were elevated respectively, was also performed. Morning surge was defined as the difference between the nighttime BP and the mean early morning BPs. Abnormal morning surge was considered above 23 mmHg.

All patients’ data were anonymized following data collection to prevent possible identification of the patients whose medical records were reviewed. The study was approved by the Rabin Medical Center institutional review board and all methods were performed in accordance with the relevant guidelines and regulations. All the participants provided written informed consent.

### Outcome assessments

The prevalence of masked hypertension in the study population was compared with the known prevalence of masked hypertension in the general population in Israel. Another goal of this study was to compare patients with AF and masked hypertension and patients with AF without masked hypertension, in order to characterize the clinical and demographic parameters of patients with AF and risk of masked hypertension.

In addition, all patients were followed for 1 year following performance of the 24 h ABPM for any new diagnoses of hypertension or hospitalization. Diagnosis of hypertension were usually based on clinic and home measuring of blood pressure.

Blinding is not relevant in this type of study and since the study is not interventional there are no adverse effects expected.

### Statistical analysis

The statistical analysis for this paper was generated using SAS Software, Version 9.4. Continuous variables were presented by Mean ± Std, Categorical variables were presented by (N, %). Normality of continuous variables was assessed graphically. T Test was used to compare the value of normally distributed continuous variables between study groups, Wilcoxon was used for non-normal continuous variables and Fisher’s exact was used to compare the value of categorical variables between study groups. In addition to the main analysis, we made a stratification analysis according to BMI above 25 and MBI 25 or lower. A multivariable analysis was constructed according to univariable analysis by entering variables that were statistically significant in the univariable analysis, as well as age and gender. Two-sided p values less than 0.05 were considered statistically significant.

## Results

### Study population

**Figure 1:**
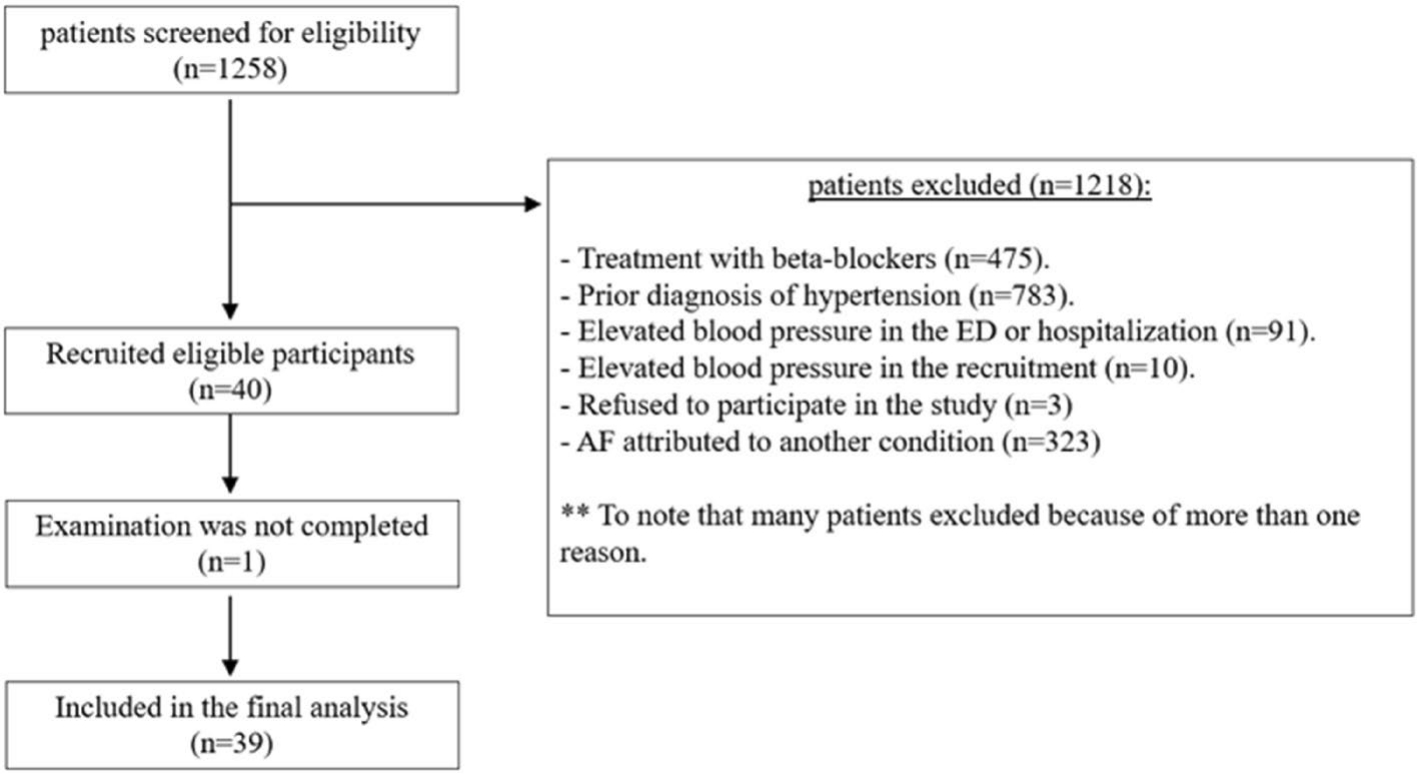
Eligibility chart.

A total of 1258 patients were screened for eligibility. Of them, 1218 patients were excluded and the reasons for exclusion are presented in Fig. 1. Forty patients were included in the final analysis, one patient didn’t complete the examination. The baseline characteristics of the study cohort are presented in Table 1. The average age was 53.4 ± 16.6 years, 28 patients (72%) were male gender, and 32 patients (82%) were healthy without any comorbidity on admission. The average BMI among the masked hypertensives group was 28.0 ± 4.5 compared to 23.3 in the normotensives group (P < 0.001). From the total cohort, 20 patients received antiarrhythmic therapy during the ED visit.

**Table 1:** Baseline characteristics of the study cohort.

		Normotensives (n = 21)	Masked hypertensives (n = 18)	All (n = 39)	P value
Demographic	Age (mean ± std)	50.5 ± 18.0	56.7 ± 14.6	53.3 ± 16.6	0.26
	Gender—male N (%)	16 (41%)	12 (31%)	28 (72%)	0.72
	Origin Israeli N (%)	17 (44%)	16 (41%)	33 (85%)	0.67
	Origin Arabic N (%)	4 (10%)	2 (5%)	6 (15%)	
Vital signs on admission (mean ± std)	Blood pressure systolic	114 ± 13	125 ± 8	119 ± 12	0.004
	Blood pressure diastolic	70 ± 10	78 ± 8	73 ± 10	0.01
	Heart rate	73.9 ± 14.8	72.9 ± 12.0	73.4 ± 13.4	0.81
Symptoms on admission N (%)	Palpitations	13 (33%)	12 (31%)	25 (64%)	1.00
	Tachycardia	9 (23%)	8 (21%)	17 (44%)	1.00
	Dyspnea	6 (15%)	6 (15%)	12 (31%)	1.00
	Dizziness	6 (15%)	3 (8%)	9 (23%)	0.46
	Weakness	9 (23%)	6 (15%)	15 (38%)	0.74
	Syncope	4 (10%)	2 (5%)	6 (15%)	0.67
ECG N (%)	*ECG changes	4 (10%)	6 (15%)	10 (26%)	0.46
Echocardiogram N (%)	Echocardiogram After the event	14 (36%)	8 (21%)	22 (56%)	0.21
	**Abnormal echo results	3 (8%)	2 (5%)	5 (13%)	0.46
Laboratory on admission (mean ± std)	Hemoglobin	14.3 ± 1.1 (n = 20)	15.2 ± 1.8 (n = 16)	14.7 ± 1.5	0.10
	Creatinine	0.9 ± 0.2 (n = 20)	1.0 ± 0.2 (n = 17)	0.9 ± 0.2	0.21
	Glucose	107.1 ± 50.4 (n = 19)	128.7 ± 37.1 (n = 17)	117.3 ± 45.3	0.16
	Natrium	141.3 ± 2.3 (n = 20)	140.7 ± 1.3 (n = 17)	141.0 ± 1.9	0.38
	Potassium	4.4 ± 0.3 (n = 19)	4.4 ± 0.4 (n = 17)	4.4 ± 0.4	0.85
	Calcium	9.4 ± 0.4 (n = 15)	9.7 ± 0.4 (n = 14)	9.5 ± 0.5	0.05
	TSH	2.2 ± 1.2 (n = 17)	2.2 ± 1.9 (n = 9)	2.2 ± 1.4	1.00
	BMI (mean ± std)	23.3 ± 3.2	28.0 ± 4.5	25.5 ± 4.5	< .001
Comorbidities N (%)	Diabetes mellitus	0 (0%)	3 (8%)	3 (8%)	0.09
	IHD	0 (0%)	1 (3%)	1 (3%)	0.46
	CHF	0 (0%)	0 (0%)	0 (0%)	
	COPD	0 (0%)	1 (3%)	1 (3%)	0.46
	CVA	0 (0%)	0 (0%)	0 (0%)	
	CKD	0 (0%)	0 (0%)	0 (0%)	
	OSA	2 (5%)	0 (0%)	2 (5%)	0.49
	Smoking status: Current Former Never	n = 19 5 (13%) 2 (5%) 12 (31%)	n = 17 3 (8%) 3 (8%) 11 (28%)	n = 36 8 (21%) 5 (13%) 23 (59%)	0.73
Hospitalizations	Hospitalizations N (%)	12 (31%)	7 (18%)	19 (49%)	0.34
	Duration of hospitalization (days) (mean ± std)	1.8 ± 2.1	1.1 ± 1.5	1.5 ± 1.5	0.30
Outcome in 1 year	*** Number of Hospitalizations	6	4	10	0.76
	Diagnosis and treatment of HTN N (%)	0 (0%)	5 (13%)	5 (13%)	0.03

TSH—thyroid stimulating hormone, BMI—Body Mass Index, IHD—Ischemic heart disease, CHF—Congestive heart failure, COPD—Chronic Obstructive Pulmonary Disease, CVA—Cerebro Vascular Accident, CKD—Chronic kidney disease, OSA—Obstructive Sleep Apnea.

*ECG changes—nonspecific ECG changes in addition to signs of atrial fibrillation.

**Abnormal echo results—abnormality in a severity of more than mild.

Normotensives:

1. EF = 45–50%.

2. Moderate TR.

3. Aortic root dilatation.

Masked hypertensives:

1. Moderate MR, mild to mod TR, moderate pulmonary HTN, EF 53%.

2. Patent foramen ovale.

*** Reasons for hospitalization in 1 year: CVA (1), AF (4), chest pain (2), malignancy (1), bradycardia (1), pulmonary congestion (1).

Blood pressure on admission is an office/clinic BP measured in the ED.

The baseline characteristics of the study cohort excluding patients with BP > 130/80 mmHg on admission is presented in Table 2. Overall, 25 patients were included in this group. The average age was 56.4 ± 16.7 years, 17 patients (68%) were male. Similar to the main analysis, the average BMI among the masked hypertensives group was higher 26.0 ± 2.5 compared to 23.2 ± 3.2 in the normotensives group (P = 0.04).

**Table 2:** Baseline characteristics of the study cohort excluding patients with BP > 130/80 on admission.

		Normotensives (n = 17)	Masked hypertensives (n = 8)	All (n = 25)	P value
Demographic	Age (mean ± std)	53.5 ± 16.7	62.8 ± 16.0	56.4 ± 16.7	0.20
	Gender – male N (%)	12 (71%)	5 (29%)	17 (68%)	1.00
	Origin Israeli N (%)	15 (68%)	7 (32%)	22 (88%)	1.00
	Origin Arabic N (%)	2 (67%)	1 (33%)	3 (12%)	
Vital signs on admission (mean ± std)	Blood pressure systolic	110.5 ± 9.8	122.3 ± 7.0	114.3 ± 10.4	0.006
	Blood pressure diastolic	67.7 ± 8.5	73.6 ± 4.2	69.6 ± 7.8	0.03
	Heart rate	69.9 ± 11.4	70.1 ± 13.1	70.0 ± 11.7	0.96
Symptoms on admission N (%)	Palpitations	12 (71%)	4 (50%)	16 (64%)	0.39
	Tachycardia	7 (41%)	5 (63%)	12 (48%)	0.41
	Dyspnea	5 (29%)	2 (25%)	7 (28%)	1.00
	Dizziness	4 (24%)	1 (13%)	5 (20%)	1.00
	Weakness	6 (35%)	2 (25%)	8 (32%)	1.00
	Syncope	2 (12%)	0 (0%)	2 (8%)	1.00
ECG N (%)	* ECG changes	3 (18%)	4 (50%)	7 (28%)	0.16
Echocardiogram N (%)	Echocardiogram After the event	11 (65%)	4 (50%)	15 (60%)	0.67
	**Abnormal echo results	2 (12%)	1 (13%)	3 (12%)	1.00
Laboratory on admission (mean ± std)	Hemoglobin	14.2 ± 1.2 (n = 16)	14.4 ± 1.8 (n = 6)	14.3 ± 1.3	0.78
	Creatinine	0.9 ± 0.2 (n = 16)	1.0 ± 0.2 (n = 7)	0.9 ± 0.2	0.12
	Glucose	98.5 ± 16.3 (n = 15)	139.6 ± 48.6 (n = 7)	111.6 ± 35.2	0.07
	Natrium	141.3 ± 2.5 (n = 16)	140.4 ± 1.6 (n = 7)	141.0 ± 2.2	0.43
	Potassium	4.4 ± 0.3 (n = 15)	4.4 ± 0.5 (n = 7)	4.4 ± 0.4	0.86
	Calcium	9.3 ± 0.4 (n = 12)	9.5 ± 0.4 (n = 6)	9.3 ± 0.4	0.29
	TSH	2.0 ± 0.9 (n = 13)	2.8 ± 3.3 (n = 3)	2.2 ± 1.5	0.73
	BMI (mean ± std)	23.2 ± 3.2	26.0 ± 2.5	24.1 ± 3.3	0.04
Comorbidities N (%)	Diabetes mellitus	0 (0%)	2 (25%)	2 (8%)	0.09
	IHD	0 (0%)	0 (0%)	0 (0%)	
	CHF	0 (0%)	0 (0%)	0 (0%)	
	COPD	0 (0%)	1 (12.5%)	1 (4%)	0.32
	CVA	0 (0%)	0 (0%)	0 (0%)	
	CKD	0 (0%)	0 (0%)	0 (0%)	
	OSA	2 (12%)	0 (0%)	2 (8%)	1.00
	Smoking status: Current Former Never	n = 16 4 (25%) 1 (6%) 11 (69%)	n = 8 2 (25%) 2 (25%) 4 (50%)	n = 24 6 (25%) 3 (13%) 15 (63%)	0.48
Hospitalizations	Hospitalizations N (%)	9 (53%)	2 (25%)	11 (44%)	0.23
	Duration of hospitalization (days) (mean ± std)	1.2 ± 1.4	0.6 ± 1.2	1.0 ± 1.3	0.29
Outcome in 1 year	*** Number of Hospitalizations	6	1	7	0.25
	Diagnosis and treatment of HTN N (%)	0 (0%)	2 (25%)	2 (8%)	0.08

TSH—thyroid stimulating hormone, BMI—Body Mass Index, IHD—Ischemic heart disease, CHF—Congestive heart failure, COPD—Chronic Obstructive Pulmonary Disease, CVA—Cerebro Vascular Accident, CKD—Chronic kidney disease, OSA—Obstructive Sleep Apnea.

* CG changes—nonspecific ECG changes in addition to signs of atrial fibrillation.

**Abnormal echo results—abnormality in a severity of more than mild.

Normotensives:

1. EF = 45–50%.

2. Aortic root dilatation.

Masked hypertensives:

1. Moderate MR, mild to mod TR, moderate pulmonary HTN, EF 53%.

*** Reasons for hospitalization in 1 year: AF (3), chest pain (2), malignancy (1), pulmonary congestion (1).

Blood pressure on admission is an office/clinic BP measured in the ED.

### Clinical outcomes

From extraction of the 24 h ABPM examination, 18 individuals (46.2%) had abnormal BP values according to the 2017 ACC/AHA guidelines for the diagnosis of hypertension^15^. Of these, 12 patients (31%) had abnormal 24-h BP average (≥ 125/75 mmHg), one patient (3%) had isolated daytime abnormal average (≥ 130/80 mmHg), 11 patients (28%) had isolated night time abnormal average (≥ 110/65 mmHg) and six patients (15%) had combined day time & night time abnormal average. 21/39 patients were normotensives (53.8%). In the analysis excluding patients with BP > 130/80 mmHg during the ED visit based on the ACC/AHA guideline, 8/25 patients (32%) were diagnosed with masked hypertension. To note that also according to the mitigating criteria of the 2018 ESC/ESH guidelines for the management of arterial hypertension^13^, the prevalence of masked HTN in our total cohort was high and was found in 11 patients (28.2%).

### Non-dipping, systolic and diastolic load, morning surge

Non-dipping and blood pressure load are present in Table 3.

**Table 3:** Mean daytime, nighttime and 24 h blood pressures. Non-dipping and blood pressure load.

	Normotensives (n = 21)	Masked hypertensives (n = 18)	All (n = 39)	P value
Mean blood pressure 24 h	117/69			
Mean daytime blood pressure	121/72			
Mean nighttime blood pressure	109/61			
Non dippers	15 patients (38%)			
Systolic load Median (min,max)				
Day (%)	2.0 (0.0,13.0)	12.5 (0.0,91.0)	6.0 (0.0,91.0)	0.006
Night (%)	6.0 (0.0,25.0)	26.0 (6.0,100.0)	12.0 (0.0,100.0)	< .001
24 h (%)	2.0 (0.0,13.0)	21.0 (2.0,90.0)	8.0 (0.0,90.0)	0.001
Diastolic load Median (min,max)				
Day (%)	2.0 (0.0,6.0)	8.5 (0.0,33.0)	4.0 (0.0,33.0)	0.001
Night (%)	0.0 (0.0,7.0)	3.0 (0.0.29.0)	0.0 (0.0,29.0)	0.020
24 h (%)	2.0 (0.0,5.0)	9.0 (0.0,25.0)	3.0 (0.0,25.0)	< .001

From the total cohort, 15 patients (38%) were non-dippers, 12 patients (31%) were extreme dippers, one patient (3%) was a reverse dipper, and only 10 patients (26%) were normal dippers. Unfortunately, to one patient the data regarding dipping was missing. From the normal dippers, three patients had a diagnosis of masked HTN and the rest were normotensives. The median of nighttime systolic blood pressure load was 26% and 6% in the hypertensive and normotensive groups respectively, P < 0.001 (Table 3). Seven patients from 19 in whom calculation of morning surge was possible (37%) had abnormal morning surge. Non-dipping and blood pressure load of the cohort of patients excluding stage 1 hypertensives patients (BP > 130/80 mmHg) on admission are presented in Table 4. From the total cohort, 9 patients (36%) were non-dippers, 8 patients (32%) were extreme dippers, one patient (4%) was a reverse dipper, and only 7 patients (28%) were normal dippers.

**Table 4:** Mean daytime, nighttime and 24 h blood pressures. Non-dipping and blood pressure load of the study cohort excluding patients with BP > 130/80 on admission.

	Normotensives (n = 17)	Masked hypertensives (n = 8)	All (n = 25)	P value
Mean blood pressure 24 h	115/67			
Mean daytime blood pressure	118/70			
Mean nighttime blood pressure	106/59			
Non dippers	9 patients (36%)			
Systolic load Median (min,max)				
Day (%)	2.0 (0.0,13.0)	12.5 (0.0,33.0)	6.0 (0.0,33.0)	0.003
Night (%)	6.0 (0.0,25.0)	37.5 (9.0,94.0)	9.0 (0.0,94.0)	< .001
24 h (%)	4.0 (0.0,13.0)	24.0 (3.0,41.0)	5.0 (0.0,41.0)	< .001
Diastolic load Median (min,max)				
Day (%)	2.0 (0.0,6.0)	9.5 (0.0,14.0)	3.0 (0.0,14.0)	0.01
Night (%)	0.0 (0.0,7.0)	0.0 (0.0.29.0)	0.0 (0.0,29.0)	0.17
24 h (%)	2.0 (0.0,5.0)	9.0 (0.0,19.0)	2.0 (0.0,19.0)	0.01

### Prevalence of masked HTN according to BMI

When stratifying data according to BMI, 21 individuals (54%) had a BMI greater than 25. Of them, 14 individuals (66%) had masked HTN and 7 patients (33%) were normotensives, P = 0.01. These results are present in Table 5.

**Table 5:** Prevalence of masked HTN according to BMI.

	Normotensives (n = 21)	Masked hypertensives (n = 18)	All (n = 39)	P value
BMI ≤ 25	14 patients	4 patients	18 patients	0.01
	78%	22%	100%	
BMI > 25	7 patients	14 patients	21 patients	
	33%	66%	100%	

*BMI* body mass index.

In a multivariable analysis for age, gender and body mass index, only high BMI was associated with the risk for masked hypertension, HR of 1.45 (CI of 1.09–1.93) (Table 6).

**Table 6:** Multivariable analysis for age, gender and body mass index.

	HR (95% CI)	P value
Age	1.007 (0.953–1.063)	0.8167
Gender	1.019 (0.58–6.551)	0.9845
Body mass index	1.45 (1.09–1.93)	0.0118

*HR* hazard ratio; *CI* confidence interval.

*For continuous variables HR given per 1 unit increment: year (for age), kg/m^2^ (for BMI).

### Follow-up period of 1 year

After a follow-up period of 1 year, five patients in the masked HTN group had a confirmed diagnosis of hypertension compared to no patients in the normotensives group, P = 0.03. In the cohort of patients excluding patients with BP > 130/80 mmHg on admission, two patients in the masked HTN group had a confirmed diagnosis of hypertension after 1 year, compared to no patients in the normotensives group, P = 0.08.

## Discussion

The prevalence of masked hypertension in our cohort of patients with lone AF and without a diagnosis of hypertension was 46.2%. This prevalence is significantly higher than the figures previously reported and is true for both the 2017 ACC/AHA and the 2018 ESC/ESH guidelines for the management of arterial hypertension.

The decision to include BP values in the range of 130/80–140/90 mmHg (excluding stage 2 hypertension according to the ACC/AHA guideline) in the main analysis was based on the assumption that non-office-based measurement may reasonably be higher in the ED and according to all guidelines, hypertension is usually diagnosed based on several elevated BP measurements and an elevated BP on a single occasion, particularly during visit to ED, cannot be used to diagnose this condition. Furthermore, often, patients with mildly elevated BP values during the ED visit (130/80–140/90 mmHg) are not followed-up as these elevated values are attributed to the stressful situation of being in the ED. Although this was not the purpose of the study, we do believe that continued follow-up of these patients is essential particularly in those with atrial fibrillation. The high prevalence of masked hypertension even after excluding patients with stage 1 hypertension during the ED visit (32%) serves as additional evidence for the importance of performing ABPM to all patients with atrial fibrillation, even when BP values are entirely within normal limits even under stressful conditions, such as during an ED visit.

Interestingly, a recently published study examining the risk of new-onset AF in patients with masked uncontrolled hypertension found that the risk is approximately doubled for patients with controlled HTN^16^. Another study evaluated patients aged ≥ 75 years with non-valvular AF and found that masked uncontrolled morning hypertension was very prevalent in these patients^17^.

ABPM is an important diagnostic tool for the diagnosis of masked hypertension in the general population and it is also a useful tool for follow-up of treated patients^18^. Given the high prevalence of masked hypertension in our population of patients with lone AF without a diagnosis of HTN, we believe that ABPM in such individuals should be strongly considered, for identification and early treatment of HTN. Early diagnosis of hypertension may significantly diminish cardiovascular risk, as has been shown in several previous studies^13,15,19^. Since masked hypertension is difficult to diagnose and since it is impractical to perform ABPM to the entire population, it is crucial to identify populations at risk for this condition, in which ABPM is cost-effective. The fact that masked hypertension was extremely prevalent in individuals with atrial fibrillation in our cohort may serve as preliminary evidence for performing ABPM in all individuals with AF. It seems that AF is prevalent in patients with masked hypertension due to the fact that elevated BP leads to left ventricular hypertrophy, left atrial enlargement and this probably increases the risk of atrial arrythmias. This has been previously reported for patients with overt hypertension^20,21^ and therefore it is not surprising that it would be true for patients with masked hypertension.

In our cohort, 41% had abnormal night-time BP average. This finding of abnormal nocturnal BP among masked hypertensives is consistent with another study from Israel^22^, that assessed ambulatory blood pressure in 4121 subjects and found that masked hypertension was more common according to the awake-sleep blood pressure method.

In our cohort, a very high rate of non-dippers was found (38%). According to the literature, normal BP fall between daytime and the nighttime is approximately 15 percent^23^. Non-dipping is the failure of BP to drop during sleep by at least 10 percent and it is associated with risk for development of heart failure, stroke and cardiovascular complications^24–28^, and with the risk of progression of nephropathy among patients with diabetes and with decline in renal function among patients with chronic kidney disease^29–32^. Moreover, in our cohort, only 26% of individuals were normal dippers. Finally, 37% of the patients in our cohort had abnormal morning surge, which was found to be associated with adverse cardiovascular outcome in elderly dippers^33^.

In a sub-analysis according to BMI, an association was found between masked HTN and overweight. Overall, 66% of patients with BMI above 25 had masked HTN, P = 0.01. Also according to multivariable analysis for age, gender and body mass index, only high BMI was associated with the risk for masked hypertension. Therefore, it seems that ABPM is especially warranted to individuals with AF who also have BMI > 25, in which masked hypertension is particularly common.

In our cohort, the average age was relatively young, and most individuals were males without comorbidities. These findings are consistent with previous studies in which masked hypertension was more common among males^34^. Young healthy males are expected to benefit significantly from the identification of masked hypertension as they have a long-life expectancy. Thus, the occurrence of AF in such individuals should alert the clinician to the possibility of masked hypertension, as identification of this condition in young healthy adults would be expected to significantly improve cardiovascular outcomes if BP would be identified and treated appropriately. It is important to note that in our cohort, comorbidities were equally prevalent in those with and without masked hypertension. This in contrast to previous studies in which masked hypertension was more prevalent in individuals with diabetes mellitus^6,30^, chronic kidney disease^31^ and obstructive sleep apnea^35^. This highlights the fact that ABPM should be performed in patients with AF, irrespective of comorbidities.

This study has several limitations. The first is the small sample size, which is due to the strict criteria used for screening and exclusion of patients who were discharged from the ED with beta blockers or calcium channel blockers, which was the main reason for exclusion. In our opinion, it was crucial to exclude such individuals because these medications may have caused a decrease in BP, leading to misidentification of these patients as normotensive. It is evident that it is difficult to identify patients according to our strict exclusion criteria but it seems that the large percentage of patients who had masked hypertension should not be influenced from the sample size. A second limitation stems from a selection bias and the homogeny of the study population. Only patients with lone AF without a diagnosis of hypertension were included and therefore the conclusions of this manuscript apply to this population. Yet, considering the large number of patients diagnosed with masked hypertension, it would be logical to assume that older individuals with more comorbidities would have a higher prevalence of masked hypertension. Additional limitation is that BP measurements in the ED were made using automated BP measurement technique, rather than auscultatory methods, which are considered more precise and are the preferred method for measuring blood pressure in patients with AF. Since most BP measurements performed in the ED are performed using automated BP devices, we believe that this information represents real world data by which patients with masked hypertension will be identified in the ED. In addition, several studies have already validated the reliability of BP monitoring devices in individuals with AF^36^.

## Conclusion

This study has clinical significance because it is one of the first studies to evaluate the prevalence of masked hypertension in patients with AF. The results certainly support performing ABPM to all patients with new onset AF, irrespective of comorbidities. Whether early identification and treatment of masked hypertension in these patients will lead to improved cardiovascular outcomes, requires long-term large-scale studies.

## Data Availability

The datasets generated and analysed during the current study are not publicly available due to privacy/ethical restrictions but are available from the corresponding author on reasonable request.
